# Radioprotective efficacy of plastic polymer against the toxicogenomic effects of radiopharmaceutical ^18^F-FDG on human lymphocytes

**DOI:** 10.1186/s13014-020-01598-0

**Published:** 2020-06-17

**Authors:** Nilson Benedito Lopes, Igor Vivian Almeida, Pedro Henrique Silvestre Lopes, Veronica Elisa Pimenta Vicentini

**Affiliations:** 1 Department of Physics, State University of Maringá, Maringá, Paraná Brazil; 2 Department of Biotechnology, Genetics and Cell Biology, State University of Maringá, Avenida Colombo, 5.790, Bloco H67, Sala 11, Jardim Universitário, Maringá, Paraná 87020-900 Brazil; 3 Federal Rural University of Amazonia, Capitão Poço, Pará Brazil; 4 Angelina Caron Hospital, Curitiba, Paraná Brazil

**Keywords:** Fluorodeoxyglucose, Radiological shielding, Increased individual protection, Human biomonitoring, Micronucleus

## Abstract

**Background:**

Healthcare workers occupationally exposed to ^18^F-FDG cannot wear protective equipment, such as lead aprons, since the interaction between high energy radiation (511 keV) and metal increases the dose of radiation absorption. The objective of this study was to evaluate the shielding efficacy of a plastic polymer against the toxicogenomic effects of ionizing radiation in human lymphocytes, using cytokinesis-block micronucleus assays.

**Methods:**

Human peripheral blood lymphocytes were isolated from three subjects and cultured under standard conditions. The cultures were exposed to 300 mCi of ^18^F-FDG at a distance of 10 cm for 10 min, in the absence of shielding or with lead, polymer, and lead + polymer shields.

**Results:**

Lead shielding was found to increase the number of counts detected by Geiger-Müller radiation monitors as a consequence of the photoelectron effect. Conversely, the lead + polymer shield reduced the number of counts. The lead, polymer, and lead + polymer shields significantly reduced the frequency of micronuclei, nucleoplasmic bridges, and nuclear buds induced by ionizing radiation. Regarding cytotoxicity, only the lead + polymer shield re-established the cell cycle at the level observed for the negative control.

**Conclusions:**

Lead aprons that are internally coated with polymer increased the radiological protection of individuals occupationally exposed to ^18^F-FDG PET/CT, especially during examinations.

## Background

Radioactive fluorodeoxyglucose (2-[^18^F]-fluor-2-deoxy-D-glucose, or ^18^F-FDG) is a radiopharmaceutical widely used in PET/CT (positron emission tomography/computed tomography) exams. ^18^F-FDG is captured by glucose transporters, abundant in neoplastic cells due to their high metabolism, but not consumed by cells and therefore remain in the cytoplasm [19]. Radioactive fluorine (^18^F) is a positron emitter with a half-life of approximately 110 min. The positron travels a short distance in tissues or water before consuming its kinetic energy and combining with an electron. The interaction, named positron annihilation, results in the simultaneous emission of two photons with both having a high specific energy (511 keV) [2] that increases the risk to occupationally exposed individuals [33].

High doses of ionizing radiation (IR) can have deleterious consequences in humans, like the development of cardiovascular disease and cataracts [6, 28, 41], in addition to producing reactive oxygen species and inducing DNA damage [27, 34, 45]. The genotoxic effects of IR can cause genomic instability and mutations that lead to the development of cancer in exposed individuals [27, 37, 43]. Professional radiation protection/shielding is an important method of protection against IR exposure. Protective devices are used in hot laboratory areas during the preparation and handling of radiopharmaceuticals. They are also used when injecting patients with radiopharmaceuticals through syringes or vial shields. These shields are typically made of lead, tungsten, or lead-coated steel. Studies have shown the unprotected radiation dose to be 10 to 20 times higher than the exposure under lead shielding [1, 26, 30, 46]. The choice of leaded or unleaded aprons, apron thickness, and durability according to the manufacturer’s warranty are all important factors to consider.

The biggest drawback of protective equipment despite the radiological protection is the heavy weight (especially of those containing lead), which can cause back pain, discomfort, and muscle fatigue, and thus reducing ergonomics. The development of new, more ergonomic shields that provide radiological protection similar to lead aprons could contribute to a solution. Polyvinyl chloride (PVC) consists of long carbon chains, where every carbon atom has a chlorine atom attached to it. PVC is one of the most widely used plastic polymers in the world. Its widespread use in industry is due to its low cost and versatility, with applications ranging from thermoplastics and thermosets, to elastomeric shapes [40].

Considering the importance of constant radiological biomonitoring in individuals occupationally exposed to IR, the cytokinesis-block micronucleus (CBMN) assay in human peripheral blood lymphocytes has become one of the most commonly used tests to measure numerical and structural chromosomal alterations in human cells in vitro and in vivo [22, 36]. This assay is a reliable test for assessing radiation-induced chromosome damage and is a valuable biomarker in many biomonitoring studies among individuals that are occupationally or environmentally exposed to IR [13, 16, 34, 37]. Thus, the objective of this study was to evaluate the radiological protection efficacy of a plastic polymer, in the presence or absence of a lead shield, against the toxicogenomic effects of ionizing radiation emitted by the radiopharmaceutical ^18^F-FDG in human lymphocytes, using the CBMN assay.

## Methods

### Measuring instruments and shields

Geiger-Müller radiation monitors (which determine the number of counts per minute), model MIR-7028 (MRA Electronic Equipment Industry, Brazil), and Inspector Alert model Nuclear Radiation (International Medcom, USA), which are often used to evaluate radiation levels in workplaces [7], were used. The monitors were calibrated to measure the equivalent dose (μSv/h) and counts per minute (CPM) rates under standard conditions, according to the National Nuclear Energy Commission (CNEN), Brazil. A benchmarking test was performed to verify the compatibility between the dose rate reading and the expected nominal value for the ^137^Cs.

Two types of shields were evaluated, the first consisting of a lead shield (1 mm), similar to that found in lead aprons, and the second of a PVC polymer shield (0.5 mm). Both were tested alone or in combination.

### Shielding test

**Fig. 1:**
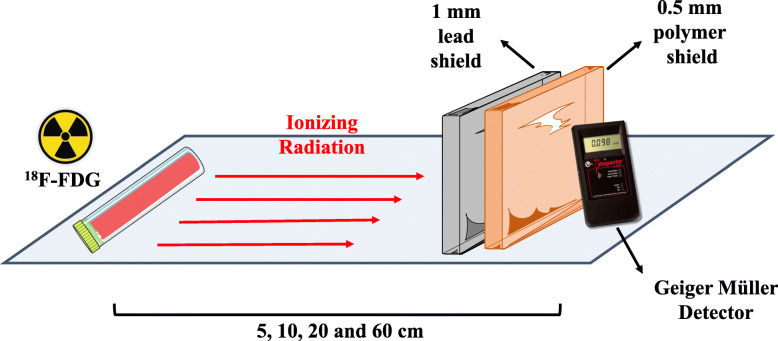
Schematic illustration of the lead + polymer shielding test

The radioactive activity of the radiopharmaceutical ^18^F-FDG (A = 4.90, 2.60, and 0.68 mCi) was measured at distances of 5, 10, 20, and 60 cm under various shield conditions next to a Geiger-Müller detector, as follows: in the absence of shielding, shielding with lead, shielding with polymer, shielding with polymer + lead, or shielding with lead + polymer. Figure 1 represents the lead + polymer shielding scheme.

### Chemicals

RPMI 1640 culture medium (R6504) and cytochalasin-B (C6762) were purchased from Sigma (USA). Fetal bovine serum (12657), phytohemagglutinin (10576), and antibiotics (penicillin and streptomycin - 15,140,122) were purchased from Gibco (USA). Giemsa dye was purchased from Merck (Germany). ^18^F-FDG (lot #FDG001686) was purchased from IBF Brazilian Pharmaceutical Industry (Brazil), which was registered and authorized for distribution of this radiopharmaceutical (CNEN 0029017459/2018). All other reagents used were obtained from laboratories in Brazil.

### Selection of volunteers

This work was carried out at the Mutagenesis Laboratory (CNEN Registry 15,012) of the Department of Biotechnology, Genetics, and Cell Biology at the State University of Maringá. All experimental procedures were approved by the Committee on Ethics in Human Research. The purpose, scope, benefits, risks, and procedures of the study were explained to each participant and informed consent was obtained from each donor. Three healthy male donors, aged 22, 28, and 30 years old, were voluntarily recruited to observe the spontaneous and induced frequencies of DNA damage (MN – micronucleus, NPB – nucleoplasmic bridge, and NBUD – nuclear bud) and cytokinesis-block proliferation index (CBPI). The subjects had no history of chronic disease, smoking, chemical abuse, or exposure to toxic substances. No radiation exposure or viral infection for 6 months before the study was documented. Peripheral blood samples (maximum of 20 mL for each donor) were collected by venipuncture and placed in heparinized tubes. Two collections were made 30 days apart.

### Lymphocyte culture and cytokinesis-block micronucleus assay

Leukocytes isolated from whole peripheral blood (500 μL) by simple decantation (3 h) were initially added to RPMI 1640 medium supplemented with fetal bovine serum (10%), phytohemagglutinin (1%), and antibiotics, and then incubated at 37 °C and 5% CO_2_ for 4 h for stabilization. After exposure to ^18^F-FDG, according to the irradiation protocol described in section 2.6, the cultures were returned to the incubator. The CBMN assay was performed as described by Fenech and Morley [12] and certified by the Organization for Economic Co-operation and Development [29]. Cytochalasin-B (6 μg/mL) was added to prevent cytoplasm division after 44 h of phytohemagglutinin stimulation, and cells were harvested within 72 h. Lymphocytes were fixed in a methanol-acetic acid solution. The slides were drip mounted, air-dried, and stained with Giemsa (5%) for 5 min.

### Irradiation of cultures

**Fig. 2:**
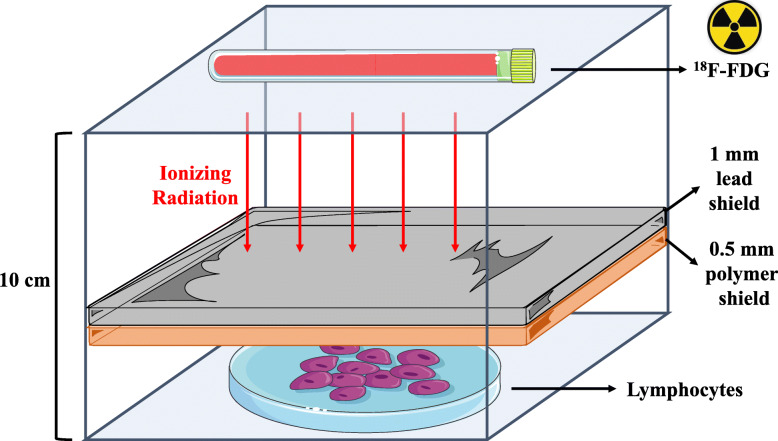
Schematic illustration of the lead + polymer shielding during exposition to ionizing radiation

Following 4 h of stabilization, the lymphocyte cultures were exposed to a glass vial containing 20 mL of 300 mCi ^18^F-FDG, at a distance of 10 cm for 10 min in an acrylic shelf-mounted device. Under these conditions, the dose absorbed by the lymphocytes’ culture was mathematically estimated at 0.06 Gy. This device intended to simulate the real-world conditions of occupational exposure (mainly for hands, forearms, and eyes during manipulation, and lower limbs during radiopharmaceutical transport and positioning of the patient for examination) in the radiology laboratory. The cultures were divided into the following groups:

- Negative control: no exposure to IR;
- ^18^F-FDG unshielded (positive control): direct exposure to IR;
- ^18^F-FDG + lead: the culture was protected with a 1-mm lead shield;
- ^18^F -FDG + polymer: the culture was protected with a 0.5-mm polymer shield;
- ^18^F -FDG + lead + polymer: the culture was simultaneously protected with a 1-mm lead shield at first, and then by 0.5 mm polymer shield (a schematic illustration is provided in Fig. 2).

### Analysis criteria

Double-blinded microscopic analysis was performed using a 400-magnification light microscope (Leica DM750). All slides were analyzed for the total number of MN, NPB, and NBUD per 1000 binucleated cells, as well as for the total number of micronucleated cells, according to the criteria previously described by Fenech [14]. The frequency of binucleated cells containing one or more MN was also determined. Only binucleated cells with a well-preserved cytoplasm were scored. The frequencies of mononucleated, binucleated, and polynucleated cells were also counted at 500 cells per individual. The CBPI was calculated on the same slides using the formula: [M1 + 2 M2 + 3 (M3 + M4)]/1000, where M1-M4 represented the number of cells with one to four nuclei, respectively, and M3 and M4 were equally considered in their third division cycle [16, 23].

### Statistical analysis

Statistical analysis was performed using the GraphPad Prism 5 software. Unpaired t-test was used to compare the number of MN, NPB, and NBUD between the different groups after irradiation. The Kolmogorov-Smirnov test was used to determine the normal distribution of the data.

## Results and discussion

As observed in the shielding test (Table 1) of the IR emitted by the ^18^F-FDG, the lead shield was not effective in reducing the number of counts registered by the radiation monitor. There was an increase in counts for all activities at all distances, except for A = 0.68 mCi, which recorded 4000 counts per minute without shielding, and 3800 with the lead shield, at 60 cm. This increase in counts was due to the photoelectric effect, which is predominant for energies less than 600 keV and chemical elements with a high atomic number, such as lead (Z = 82) [5, 39]. When the IR reaches the lead shield, the transfer of energy from the ionization electron to the material medium produces a proportional secondary ionization that expresses the incident radiation energy at the end of the process. Due to this increase in counts recorded by the radiation monitor behind the shield, it is expected that the individual exposed to the ^18^F-FDG, for example, will not use any type of lead protection equipment when handling radiopharmaceuticals, increasing exposure to IR. The polymer + lead shield fluctuated in the increase and decrease of the counts. The polymer and lead + polymer shields reduced the counts for all the radioactive activities and distances analyzed, highlighting the novelty of this study. Previous experiments showed a similar efficacy of 0.5 mm and 1 mm polymer thickness (data not presented).

**Table 1:** Absolute values (counts per minute ✕ 1000) measured in a Geiger-Müller detector for the radiopharmaceutical ^18^F-FDG, with lead and polymer shields

^**18**^F-FDG	Distance	Unshielded	Lead	Polymer	Polymer + Lead	Lead + Polymer
**A =** **4.90** **mCi**	*5 cm*	sat	sat	sat	sat	sat
	*10 cm*	sat	sat	sat	sat	sat
	*20 cm*	225	229,9	185	226,8	171,9
	*60 cm*	30	34,8	27,4	31,8	24,7
**A =** **2.60** **mCi**	*5 cm*	sat	sat	sat	sat	sat
	*10 cm*	sat	sat	266	sat	252
	*20 cm*	134	142	106,2	134	96,8
	*60 cm*	26,8	28,4	21,2	27	20,8
**A =** **0.68** **mCi**	*5 cm*	163	176	130,2	172,4	121,3
	*10 cm*	89	90,4	73,3	86	65,1
	*20 cm*	34	38	25,3	35	22,6
	*60 cm*	4	3,8	3,8	3,8	3,5

A: radioactive activity of the element. Sat: saturation of the monitor, making measurement impossible

These findings support the simulation performed by Fonsêca et al. [15]. Using the Monte Carlo N-Particle method, they demonstrated that the use of a 0.5-mm thick lead apron increased the dose absorbed by the individual exposed to IR of the ^18^F-FDG by up to 26%, instead of a reduction, depending on the distance between the radiation source and the individual. This increase occurred due to the generation of secondary and scattered particles from the interaction of the incident photons (511 keV) with the lead apron, which would not exist without the apron. Consequently, these new ionizations led to an increased flow of photons and electrons that increased the dose absorbed by the individual.

**Fig. 3:**
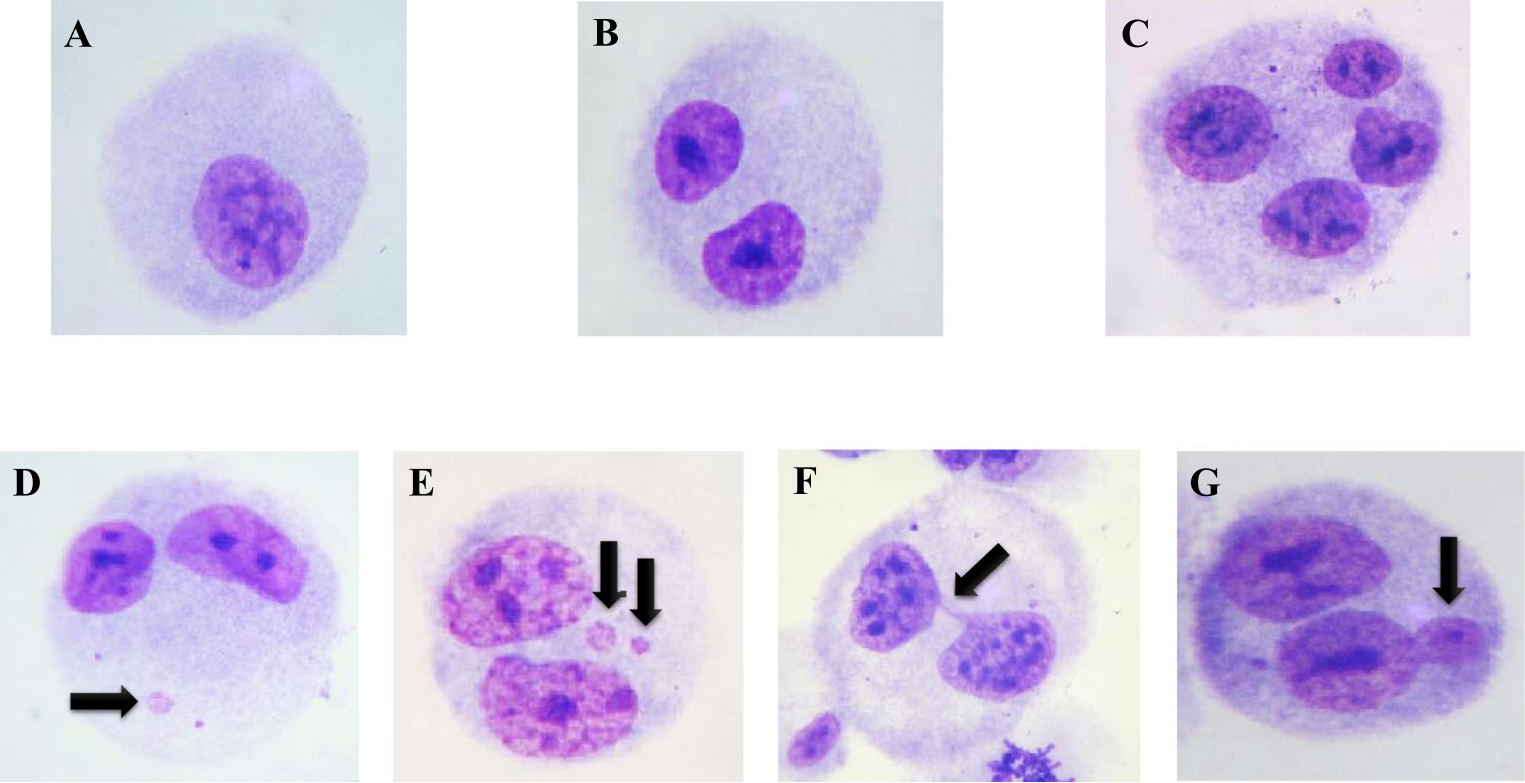
Cell proliferation and DNA-induced damage in human peripheral blood lymphocytes. **a** mononucleated cell. **b** binucleated cell. **c** polynucleated cell. **d** binucleated cell with one micronucleus. **e** binucleated cell with two micronuclei. **f** binucleated cell with nucleoplasmic bridge. **g** binucleated cell with nuclear bud. Magnification: 1000✕

The CBMN assay results (Table 2) indicated a statistically significant increase in the frequency of MN in binucleated cells (*p* < 0.0001), compared to the negative control, where the lymphocyte cultures were exposed to the ^18^F-FDG radiopharmaceutical without shielding. Shielding significantly reduced the MN frequencies (lead *p* = 0.0001, polymer *p* = 0.0133, and lead + polymer *p* < 0.0001) compared to the unshielded group. Similarly, for the NPB and NBUD frequencies, a higher induction of DNA damage was observed in cultures exposed to IR (*p* < 0.0001 and *p* = 0.0026, respectively) compared to the negative control. The polymer shield differed from the negative control (NPB *p* = 0.0171 and NBUD *p* = 0.0442), despite reducing the incidence of damage (NPB *p* = 0.0001 and NBUD *p* = 0.0108). The lead shield (NPB *p* = 0.6877 and NBUD *p* = 0.4033) and the lead + polymer shield (NPB *p* = 0.1773 and NBUD *p* > 0.9999) significantly reduced IR-induced damage to the level observed in the negative control (Fig. 3).

**Table 2:** Induction of micronuclei by ionizing radiation of ^18^F-FDG in human lymphocytes in vitro

Shielding	Distribution of BNC according to the number of MN				MN/BNC ± SD (%)	CMN/BNC ± SD (%)	NPB/CBN ± SD (‰)	NBUD/CBN ± SD (‰)	CBPI ± SD
	*1*	*2*	*3*	*4*					
Negative Control	14	1	0	0	1.55 ± 0.19	1.48 ± 0.18	1.00 ± 0.63	0.83 ± 0.75	2.09 ± 0.05
^18^F-FDG Unshielded	53	3	2	2	7.13 ± 0.70^a^	5.92 ± 0.68^a^	5.83 ± 0.75^a^	4.67 ± 1.75^a^	1.60 ± 0.06^a^
^18^F-FDG + Lead	39	2	1	1	4.78 ± 0.33^ab^	4.22 ± 0.19^ab^	1.17 ± 0.75^b^	0.50 ± 0.55^b^	1.99 ± 0.03^ab^
^18^F-FDG + Polymer	46	3	1	1	6.02 ± 0.20^ab^	5.10 ± 0.14^ab^	2.50 ± 1.05^ab^	1.83 ± 0.75^ab^	1.88 ± 0.04^ab^
^18^F-FDG + Lead + Polymer	38	1	1	0	4.30 ± 0.23^ab^	3.98 ± 0.23^ab^	1.50 ± 0.55^b^	0.83 ± 0.75^b^	2.06 ± 0.04^b^

Number of binucleated cells analyzed for each individual in each repetition = 1000

*BNC* binucleated cells, *MN* micronucleus, *CMN* cell with one or more micronuclei, *NPB* nucleoplasmic bridge, *NBUD* nuclear bud, *CBPI* Cytokinesis-Block Proliferation Index

^a^ Statistically significant difference from the negative control (*p* < 0.05)

^b^ Statistically significant difference from the unshielded group (*p* < 0.05)

The increase in the frequency of MN, NPB, and NBUD in lymphocytes reflects the chromosomal instability induced by IR. MN can be formed during anaphase when an acentric chromosome, or chromatid fragments from DNA repair failures are not incorporated into the cell’s main nucleus. NPB instead originates from dicentric chromosomes, which can occur due to the inadequate repair of breaks in DNA or terminal fusions of telomeres. NBUD represents the process of eliminating amplified DNA, complex DNA repair mechanisms, possible chromosomes of aneuploid cells, and may also result from NPB disruption [9–11, 16, 22, 36]. The direct interaction of IR with DNA induces single and double-strand breaks, changes in nitrogen bases, and DNA cross-linking [27, 45]. The genotoxic effects of IR may cause genomic instability and mutations that lead to cancer induction in exposed individuals [27]. Several studies have reported increased levels of chromosomal aberrations in lymphocytes from workers occupationally exposed to IR, compared to those from unexposed workers [3, 8, 21, 25, 35, 42, 47]. Additionally, some epidemiological studies have revealed that individuals who are occupationally exposed to IR may have an increased risk of developing leukemia and other cancers [4, 18, 20, 38, 44].

The CBPI analysis (Table 2) is a useful tool to understand the cell cycle kinetics of lymphocyte cultures, especially after exposure to IR [32, 36]. Evaluation of this parameter in the present study indicated that direct exposure to IR had a cytotoxic effect on lymphocytes, compared to the negative control (*p* < 0.0001). The polymer (*p* < 0.0001) and lead (*p* = 0.0031) shields were not effective in protecting lymphocytes from the cytotoxic effects of radiation, compared to the negative control. Only the lead + polymer shield was effective in protecting the cells, since the CBPI of this group did not present a statistically significant difference compared to the negative control (*p* = 0.2903). The results of the CBMN assay are consistent with the CBPI results, suggesting that the lead + polymer shield was equally effective in protecting the DNA and in maintaining the integrity of the lymphocytes exposed to ^18^F-FDG.

IR generates an explosion of free radicals capable of inducing DNA damage in exposed cells, because of the ionization of water molecules and the direct ionization of target molecules [24, 37]. These free radicals are produced in microseconds. However, their effects persist long after their production, owing to the cascade of events triggered at the molecular and cellular level. This ultimately leads to increased oxidative stress, lipid peroxidation, and genome instability [17, 37]. All these mechanisms can be explained by the cytotoxic and genotoxic effects observed and reaffirms the uniqueness of this study.

Considering the analyzed population (*n* = 3 individuals, 6000 cells for CBMN assay, and 3000 cells for CBPI, in each group), it is essential to consider that results with more blood donors could provide even more relevant observations on interindividual and temporal differences. It is also noteworthy that a more significant number of samples would result in a longer exposure time of researchers to ^18^F-FDG, even with the adoption of all security protocols. Moreover, the harmful effect of IR is not limited to lymphocytes. The radiosensitivity of healthy cells, tissues, and organs depend on several factors, including the ability to repair the damage, hypoxia, cell cycle position, and the effective absorbed dose of radiation. Then, further investigations with different cell types must be carried out.

Thus, compliant with the basic guidelines of CNEN (NN 3.01 and 3.05) which aim to optimize radiological protection to provide occupationally exposed individuals with better health care, we found that the use of a lead + polymer shield is more effective than lead aprons. The association of lead + polymer reduces the number of counts and mitigates the biological effects of IR, increasing the protection of individuals working in PET/CT procedures with ^18^F-FDG. Similar to the PVC polymer, Prokhorenko et al. [31] demonstrated that polystyrene composite (19.1%) combined with tungsten (66.9%) and aluminum (14%) was also effective in the production of radioprotective equipment of low weight and high durability, supporting our results.

The results from this study show that the addition of a lightweight, low-cost polymer layer to a lead apron is highly recommended and provides enhanced radiological and biological protection to individuals who are occupationally exposed to radiation, particularly during ^18^F-FDG handling. In cases where lead aprons are avoided due to their low ergonomics, we recommended the use of polymer aprons by qualified service personnel at least. Other polymer-based safety equipment may be developed to reduce the biological effects of IR, like glasses and other types of eye protection, and polymer-coated syringe/vial holders and coatings for radiopharmaceuticals. Furthermore, a minimum distance of 25 cm between healthcare professionals and radiopharmaceuticals is recommended during transportation from hot laboratories to patients’ rooms. Workers occupationally exposed to ionizing radiation who are better protected will be able to carry out routine activities safely, allowing them to work longer with less radiation exposure, reducing staff turnover costs in addition to minimizing their risk of exposure to the harmful effects of IR.

## Conclusions

Ionizing radiation is mutagenic and known to induce cell damage, including the formation of micronuclei, nucleoplasmic bridges, and nuclear buds. The results presented in this study suggest that the coating of conventional radiation shielding equipment, like lead aprons, with a polymer layer could increase the radiological protection of occupationally exposed individuals, particularly during ^18^F-FDG PET/CT examinations. The toxicogenomic biomonitoring of workers exposed to ionizing radiation, the application of radiation protection programs, and physical dosimetry procedures are all crucially important in minimizing occupational radiation exposure. Moreover, the development of new protective equipment is key in terms of biotechnology for the protection of individuals and the environment. Better protective equipment will increase the safety of health workers exposed to radiation, and also that of researchers, technicians, and students exposed to radiation in universities, research centers, and industries.

## Supplementary information

**Additional file 1.**

## Data Availability

Not applicable.

## Abbreviations

^18^F-FDG: Fluorodeoxyglucose
BNC: Binucleated cells
CBMN: Cytokinesis-Block Micronucleus
CBPI: Cytokinesis-Block Proliferation Index
CMN: Cell with one or more micronuclei
CNEN: National Nuclear Energy Commission, Brazil
CPM: Counts per minute
IR: Ionizing radiation
MN: Micronucleus
NBUD: Nuclear bud
NPB: Nucleoplasmic bridge
PET/CT: Positron Emission Tomography/Computed Tomography
PVC: Polyvinyl chloride
