# Supplementary material for: Radioprotective efficacy of plastic polymer against the toxicogenomic effects of radiopharmaceutical 18F-FDG on human lymphocytes
Source: Radiat Oncol. 2020 Jun 17;15:154. doi: 10.1186/s13014-020-01598-0 (PMC7301467; doi:10.1186/s13014-020-01598-0)
Supplement: Supplementary file 1 — Additional file 1. [file 13014_2020_1598_MOESM1_ESM.docx]

**Supplementary Table 1:** Data for each blood donor (blood sampling 1 and 2). One thousand analyzed cells.

| **Shielding** | **Blood Donor** | **Blood Sampling** | **Distribution of BNC according to the number of MN** | | | | **Total MN** | **Total NPB** | **Total NBUD** | **CBPI**  **(500 cells)** | | | |
| --- | --- | --- | --- | --- | --- | --- | --- | --- | --- | --- | --- | --- | --- |
|  |  |  | *1* | *2* | *3* | *4* |  |  |  | *M1* | *M2* | *M3* | *M4* |
| **Negative Control** | 1 | *1* | 16 | 1 | 0 | 0 | 18 | 1 | 2 | 74 | 313 | 81 | 32 |
|  |  | *2* | 14 | 0 | 0 | 0 | 14 | 0 | 1 | 69 | 301 | 93 | 37 |
|  | 2 | *1* | 13 | 1 | 0 | 0 | 15 | 1 | 1 | 75 | 295 | 102 | 28 |
|  |  | *2* | 16 | 0 | 0 | 0 | 16 | 1 | 0 | 80 | 340 | 58 | 22 |
|  | 3 | *1* | 11 | 1 | 0 | 0 | 13 | 2 | 0 | 67 | 300 | 101 | 32 |
|  |  | *2* | 15 | 1 | 0 | 0 | 17 | 1 | 1 | 63 | 322 | 90 | 25 |
| **^18^F-FDG Unshielded** | 1 | *1* | 49 | 3 | 1 | 2 | 66 | 5 | 3 | 231 | 207 | 47 | 15 |
|  |  | *2* | 43 | 2 | 2 | 2 | 61 | 6 | 4 | 220 | 218 | 50 | 12 |
|  | 2 | *1* | 60 | 3 | 2 | 2 | 80 | 6 | 5 | 257 | 189 | 38 | 16 |
|  |  | *2* | 59 | 4 | 2 | 1 | 77 | 5 | 4 | 259 | 203 | 24 | 14 |
|  | 3 | *1* | 54 | 2 | 1 | 3 | 73 | 6 | 8 | 263 | 197 | 23 | 17 |
|  |  | *2* | 51 | 3 | 2 | 2 | 71 | 7 | 4 | 260 | 205 | 22 | 13 |
| **^18^F-FDG + Lead** | 1 | *1* | 36 | 2 | 2 | 1 | 50 | 1 | 1 | 93 | 316 | 67 | 24 |
|  |  | *2* | 41 | 2 | 1 | 0 | 48 | 2 | 0 | 96 | 285 | 90 | 29 |
|  | 2 | *1* | 39 | 1 | 1 | 0 | 44 | 2 | 0 | 105 | 302 | 71 | 22 |
|  |  | *2* | 38 | 1 | 1 | 0 | 43 | 1 | 1 | 114 | 281 | 85 | 20 |
|  | 3 | *1* | 42 | 1 | 1 | 1 | 51 | 0 | 1 | 128 | 266 | 82 | 24 |
|  |  | *2* | 38 | 2 | 1 | 1 | 49 | 1 | 0 | 103 | 291 | 91 | 15 |
| **^18^F-FDG + Polymer** | 1 | *1* | 45 | 3 | 1 | 2 | 62 | 4 | 3 | 105 | 321 | 55 | 19 |
|  |  | *2* | 44 | 2 | 2 | 1 | 58 | 2 | 2 | 112 | 330 | 38 | 20 |
|  | 2 | *1* | 47 | 3 | 1 | 1 | 60 | 3 | 1 | 118 | 324 | 40 | 18 |
|  |  | *2* | 46 | 2 | 2 | 1 | 60 | 1 | 2 | 121 | 328 | 36 | 15 |
|  | 3 | *1* | 48 | 2 | 1 | 2 | 63 | 2 | 2 | 128 | 332 | 26 | 14 |
|  |  | *2* | 45 | 3 | 1 | 1 | 58 | 3 | 1 | 130 | 300 | 49 | 21 |
| **^18^F-FDG + Lead + Polymer** | 1 | *1* | 33 | 2 | 1 | 0 | 40 | 2 | 1 | 73 | 314 | 85 | 28 |
|  |  | *2* | 38 | 1 | 1 | 0 | 43 | 1 | 0 | 78 | 297 | 96 | 29 |
|  | 2 | *1* | 36 | 1 | 1 | 0 | 41 | 1 | 2 | 83 | 292 | 101 | 24 |
|  |  | *2* | 40 | 1 | 1 | 0 | 45 | 2 | 1 | 86 | 334 | 62 | 18 |
|  | 3 | *1* | 37 | 1 | 1 | 1 | 46 | 1 | 0 | 85 | 287 | 103 | 25 |
|  |  | *2* | 41 | 1 | 0 | 0 | 43 | 2 | 1 | 91 | 298 | 92 | 19 |

BNC: binucleated cells; MN: micronucleus; NPB: nucleoplasmic bridge; NBUD: nuclear bud; CBPI: Cytokinesis-Block Proliferation Index.
